# Exploring Clinical Predictors of Severe Human Metapneumovirus Respiratory Tract Infections in Children: Insights from a Recent Outbreak

**DOI:** 10.3390/microorganisms12040641

**Published:** 2024-03-23

**Authors:** Airin Veronese, Tina Uršič, Simona Bizjak Vojinovič, Jasna Rodman Berlot

**Affiliations:** 1 Department of Paediatric Pulmonology, University Children’s Hospital, University Medical Centre Ljubljana, 1000 Ljubljana, Slovenia; 2 Institute of Microbiology and Immunology, Faculty of Medicine, University of Ljubljana, 1000 Ljubljana, Slovenia; 3 Department of Infectious Diseases, University Medical Centre Ljubljana, 1000 Ljubljana, Slovenia; simona.bizjakvojinovic@kclj.si; 4 Faculty of Medicine, University of Ljubljana, 1000 Ljubljana, Slovenia

**Keywords:** human metapneumovirus, respiratory tract infection, oxygen therapy, wheezing, atopy, asthma, children

## Abstract

Human metapneumovirus (hMPV) is an important pathogen that causes both upper (URTIs) and lower respiratory tract infections (LRTIs) in children. The virus can be implicated in severe bronchiolitis and pneumonia, necessitating hospitalization, with certain cases requiring intensive care unit intervention. As part of a retrospective observational study, we aimed to identify indicators of severe hMPV respiratory tract infections in children referred to the University Children’s Hospital Ljubljana and the Department of Infectious Diseases Ljubljana, Slovenia, during a recent outbreak. We analyzed clinical data from November 2022 to January 2023 and compared the characteristics of children presenting with URTIs and LRTIs. We also examined the characteristics of children with hMPV LRTIs, distinguishing between children with and without LRTI-associated hypoxemia. Of 78 hMPV-PCR-positive pediatric patients (mean age 3.1 years; 60.3% boys), 36% had a URTI, and 64% had an LRTI. Hospitalization was required in 64% (50/78), with 42% (21/50) requiring oxygen therapy. LRTI-associated hypoxemia was more common in patients with atopy who showed dyspnea, tachypnea, crackles, and wheezing on lung auscultation. In a multivariable logistic regression analysis, wheezing detected on lung auscultation was a significant predictive factor for hypoxemic hMPV-LRTI. Specifically, children presenting with wheezing were found to be ten times more likely to experience hypoxemia. Prematurity and chronic conditions did not influence the presentation or severity of hMPV infection. This study highlights wheezing and atopy as crucial indicators of severe hMPV LRTI in children, emphasizing the importance of early recognition and intervention.

## 1. Introduction

Human metapneumovirus (hMPV) is a negative-sense single-stranded RNA virus belonging to the Paramyxoviridae family [1]. Discovered in the Netherlands in 2001, hMPV was originally isolated from children with clinical symptoms resembling human respiratory syncytial virus (RSV) infection. HMPV can cause upper respiratory tract infections (URTIs) and lower respiratory tract infections (LRTIs) in people of all ages, especially in young children and the elderly [2]. Serological studies show that high seropositivity is common in children before the age of five [3,4,5,6].

HMPV infections in some children may result in severe bronchiolitis and pneumonia, necessitating hospitalization, intensive care unit (ICU) admission, and intubation [3,7,8]. In addition, hMPV is associated with severe disease in people with asthma and chronic obstructive pulmonary disease (COPD) [9,10]. However, the factors that contribute to the varying clinical outcomes of hMPV infections remain elusive, prompting a need for comprehensive research.

HMPV is distributed worldwide, and in temperate regions, its seasonal spread generally follows the epidemic peak of RSV and influenza viruses in the late winter and spring months [1]. Phylogenetic analysis of HMPV has demonstrated the existence of two main genetic lineages termed subtypes A and B, containing within them the subgroups A1/A2 and B1/B2, respectively. Genotyping based on sequences of the F and G genes showed that subtype B was associated with increased cough duration and general respiratory systems compared to HMPV-A [11].

A recent outbreak of HMPV in our hospital settings provided a unique and timely opportunity for an observational study. The aim of this study was to investigate the clinical features of hMPV infections and identify potential indicators that can predict the development of severe respiratory tract infections (RTIs) caused by hMPV in children. By closely examining this outbreak’s patterns and clinical features, our research aimed to provide valuable insights into the understanding and treatment of hMPV infections, particularly those leading to severe respiratory complications. These findings could guide the development of future preventive measures and innovative treatment strategies.

## 2. Materials and Methods

### 2.1. Study Subjects

Children with clinical signs of acute RTI, presenting with URTI or LRTI, referred to the University Children’s Hospital Ljubljana and the Department of Infectious Diseases Ljubljana between November 2022 and January 2023 were tested for respiratory viruses. These hospitals are the largest children’s hospitals in Slovenia and served a population of 2,116,972 inhabitants during the study period. All individuals under 18 years of age who were PCR-positive for hMPV from nasopharyngeal swab samples (NPSs) were identified from a laboratory database and included in this study.

### 2.2. Study Design

A retrospective observational study was conducted to identify indicators of severe hMPV RTIs in children. Data on age, gender, comorbidities affecting disease severity, disease presentation, symptom duration at referral, lung auscultation, inflammatory biomarkers, radiographic findings, hospitalization details, length of hospital stay, need for oxygen therapy, complications, and treatment data were collected for all patients.

LRTI diagnosis was based on pathologic auscultation of the lungs and radiologic findings suggestive of LRTI. Hypoxemia was characterized as the inability to sustain hemoglobin saturation with oxygen, as measured by a pulse oximeter, equal to or greater than 92% while breathing room air. Asthma diagnoses were determined per guidelines provided by the European Respiratory Society [12]. Atopy was defined based on the criteria set forth by the American Academy of Allergy, Asthma and Immunology. This definition identifies atopy as a genetic predisposition toward developing allergic conditions such as allergic rhinitis, asthma, and atopic dermatitis.

Epidemiologic and clinical data were compared between patients with URTI and LRTI symptoms. In addition, the characteristics of children with hMPV infection with and without LRTI-associated hypoxemia were compared.

This observational study, using only routinely collected data during outpatient clinic visits, was considered low-risk, and ethical approval by the National Medical Ethics Committee was waived. Patient identities were anonymized through encrypted number codes. This study adhered to the ethical principles outlined in the Declaration of Helsinki, the Oviedo Convention on Human Rights and Biomedicine, and the Slovene Code of Medical Deontology.

### 2.3. Methods

Patients’ NPSs underwent multiplex PCR using the Respiratory Viruses 16-Well Assay V.19 (AusDiagnostics, Mascot, Australia) to detect viral infections, including respiratory syncytial virus (RSV), influenza virus A/B (Flu A/B), parainfluenza virus 1–4 (PIV 1–4), human bocavirus 1 (HBoV1), adenovirus (AdV), hMPV, human rhinovirus (HRV), enterovirus (EV), seasonal human coronaviruses (HCoV-HKU1, HCoV-NL63, HCoV-OC43, and HCoV-229E), and SARS-CoV-2 [13].

### 2.4. Analysis

Continuous variables are presented as mean (SD) or median (IQR) where appropriate. Categorical variables are described with counts and percentages. Continuous variables were compared using the independent samples *t*-test or the Mann–Whitney U-test, where appropriate. Categorical variables were compared using the Pearson chi-square test. A multiple logistic regression analysis was performed to predict hMPV LRTI requiring oxygen therapy based on epidemiological and clinical features. Statistical analysis was conducted using IBM SPSS Statistics (Version 28.0) with significance set at *p* < 0.05.

## 3. Results

**Figure 1:**
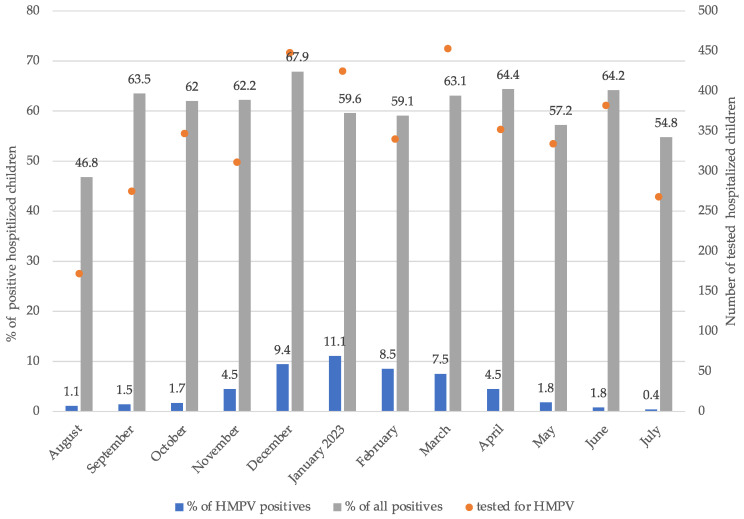
HMPV prevalence among hospitalized children between August 2022 and July 2023.

We observed a heightened prevalence of hMPV infections among hospitalized children in our medical facilities from November 2022 to April 2023 which reached its peak in January 2023 (Figure 1). In the study period, between November 2022 and the end of January 2023, 1915 children < 18 years old were hospitalized due to respiratory infections. At least one viral pathogen was detected in 63.4% (1214/1915) of patients. HMPV was detected as a single pathogen or in codetection in the NPSs of 8.4% (103/1214). After applying the study inclusion criteria, data from 78 children (mean age, 3.1 years; SD, 3.0 years; 60.3% boys) were analyzed.

Among these individuals, 20.5% (16/78) presented with comorbidities. Specifically, 5% (4/78) had nephrological disorders, 4% (3/78) had cardiac conditions, and pulmonary, neurologic, and rheumatologic disorders were each identified in 3% (2/78) of our sample. Additionally, 1% (1/78) had hematologic and dermatologic chronic diseases. Hospital admission was required for 64% of children (50/78).

Viral co-infection was identified in 42% (33/78) of cases, with HRV being the most prevalent at 23% (18/78), followed by AdV at 8% (6/78), HBoV1 at 4% (3/78), Flu A at 3% (2/78), and 1% (1/78) each for RSV, PIV, Flu B, and SARS-CoV-2, respectively.

Among the patients, 36% (28/78) exhibited signs of URTI, while 64% (50/78) manifested LRTI. Within the LRTI subgroup, acute bronchiolitis was observed in 44% (22/50), viral pneumonia in 28% (14/50), bronchitis in 18% (9/50), and asthma attack in 10% (5/50). A comparative analysis of characteristics between children presenting with hMPV PCR-positive infections as URTI or LRTI is summarized in Table 1. Notably, patients with LRTI were younger and exhibited higher C-reactive protein levels. Patients with URTI were more commonly admitted for urinary tract infections (UTIs) requiring parenteral antibiotic treatment. In contrast, patients with LRTI were predominantly admitted due to dehydration and hypoxemia, leading to more prolonged hospitalization. Interestingly, those with pre-existing chronic conditions more frequently presented with URTI and were often referred for routine check-ups due to febrile illnesses and exacerbations of their chronic conditions.

Among the children diagnosed with hMPV LRTI, 42% (21/50) required oxygen therapy. In our comparative analysis of characteristics between those with and without LRTI-associated hypoxemia, no distinctions in epidemiological features were identified (Table 2). Nevertheless, children exhibiting LRTI-associated hypoxemia displayed a higher frequency of atopy, accompanied by symptoms such as dyspnea, tachypnea, and bilateral crackles upon lung auscultation. Not surprisingly, they experienced a heightened rate of hospital admission and a more frequent administration of systemic steroids. Almost a quarter of the children with LRTI-associated hypoxemia presented with asthma.

We found no significant association between comorbidities, prematurity, and the severity of LRTI. Notwithstanding, two cases required admission to the intensive care unit (ICU). The first case involved a 1-year-old male with obstructive bronchiolitis, co-infected with Flu B, and no underlying comorbidities. The primary reason for ICU admission was hypercapnic respiratory insufficiency, necessitating non-invasive ventilation with CPAP. In the second case, a 7-year-old male presented with unilateral crackles and pneumococcal pneumonia, requiring ICU admission for pleural drainage due to empyema.

Upon further examination of children with LRTI hMPV monoinfection, our analysis revealed that bilateral crackles and wheezing were more prevalent during lung auscultation in the hypoxemic group (Table 3). Additionally, there was a higher frequency of patients presenting with an asthma attack in the hypoxemic group.

In multivariable logistic regression analysis, wheezing detected on lung auscultation emerged as a significant predictive factor for hypoxemic LRTI hMPV monoinfection (Table 4). Notably, children presenting with wheezing were found to be ten times more likely to experience hypoxemia.

Among the 21 patients presenting with wheezing upon lung auscultation at referral, 52% (11/21) necessitated oxygen therapy. Fourteen percent (3/21) were identified as atopic. Within the subset of patients presenting with an asthma attack, four out of the five had regular follow-ups in the outpatient asthma clinic before admission, with three of them being atopic. The fifth patient, while showing a robust clinical response to bronchodilator and steroid treatment for hMPV obstructive monoinfection, tested negative in allergy skin prick testing. Consequently, the diagnosis of asthma remained unconfirmed through provocation testing.

## 4. Discussion

We conducted a retrospective observational study to identify indicators of severe hMPV infection in children referred to the University Children’s Hospital Ljubljana and the Department of Infectious Diseases Ljubljana during a recent outbreak observed in our hospital. Our analysis focused on the most common clinical features of hMPV infection to identify the discernible factors contributing to clinical deterioration requiring hospitalization and the severity of LRTI. Hospitalization was mainly required in patients with hMPV-LRTIs, mainly due to hypoxemia and dehydration. Among the hospitalized patients with URTI, half had an underlying chronic disease that often required referral due to its worsening, with some cases requiring admission due to UTI and dehydration. Children with LRTI-associated hypoxemia had a higher prevalence of atopy, along with symptoms such as dyspnea, tachypnea, and bilateral crackles on lung auscultation. Not surprisingly, this group had a higher rate of hospitalization and a more frequent administration of systemic steroids. Notably, nearly a quarter of children with LRTI-associated hypoxemia presented with asthma. In a multivariable logistic regression analysis, obstructive auscultation during examination proved to be a significant predictive factor for hypoxemic LRTI caused by hMPV monoinfection. Remarkably, children who presented with wheezing were ten times more likely to develop hypoxemia.

The clinical presentations of hMPV infections observed in our patient group were consistent with the existing literature and included fever, cough, rhinitis, dyspnea, malaise, wheezing, and feeding difficulties [1]. Bronchiolitis and pneumonia are usually the leading causes of hospitalization [14,15]. In our cohort of patients, we observed similar admission patterns, but some children presented with asthma exacerbation, while others also required intervention for URTI, necessitating symptomatic and antibiotic treatment. In an American cohort, patients testing positive for hMPV and presenting with severe bronchiolitis and pneumonia required ICU admission in 6% of cases and intubation in 4% [7]. Although our tertiary facility is the primary center for treatment of the most severe RTIs in our country, our cohort exhibited a lower incidence, with only two patients (4%) requiring ICU admission and none necessitating invasive ventilation.

Previous studies have identified various factors as high-risk contributors to severe disease and hospitalization in hMPV-infected children, including prematurity, chronic diseases such as pulmonary, cardiac, renal, and immunodeficiency diseases, as well as cancer and sickle cell anemia [6]. In addition, age under five months or above 65 years and co-infection with other pathogens (especially RSV) are considered significant risk factors [1,7,8]. Interestingly, our study found no significant association between comorbidities, prematurity, and the severity of LRTI. Additionally, a previous study suggests a higher risk of LRTI in males [16], whereas we found no effect of gender on the presentation of hMPV infection.

A recent study found that hMPV infection is almost always associated with co-infection with another virus [17]. Understanding viral co-infection is critical due to its potential impact on the severity of LRTI. In our cohort, the most common viral combination was with HRV, which was detected in almost a quarter of co-infected patients. However, in a previous study, RSV, flu, and PIV were the most common viruses in combination with hMPV [16]. Interestingly, we observed no difference in the rate of viral co-infections between patients with and without hMPV LRTI-associated hypoxemia in our group, suggesting that viral co-infections had no impact on the severity of hMPV-LRTI. Consistent with our findings, previous studies also reported no differences in disease severity between children with hMPV mono- or co-infection [6,16]. However, some authors have described severe bronchiolitis in RSV/hMPV co-infection [6].

In our cohort, patients with wheezing detected on lung auscultation were ten times more likely to experience hypoxemia. In addition, atopy and asthma exacerbations were more frequently observed in children with hypoxemic hMPV-LRTI. They were also more likely to be administered systemic steroids. The association between hMPV infection and asthma remains unclear [18,19]. Definitive diagnosis of asthma in infancy, when virus-induced wheezing is common, is a major challenge. Moreover, some viruses can trigger acute inflammation and wheezing without causing chronic inflammation and asthma. Nevertheless, existing data and parallels with RSV and HRV suggest a possible link between hMPV and asthma exacerbations [9,18,20]. Studies on children and adults hospitalized for wheezing and asthma exacerbations have identified hMPV in many cases [21,22,23,24]. In addition, an earlier study concluded that hMPV bronchiolitis in infancy strongly correlates with asthma or other bronchial obstructive diseases in the third and fifth year of life [9]. In that study, hMPV was highlighted as the most important risk factor for asthma in preschool age, followed by RSV bronchiolitis and allergic rhinitis. In our study, most patients who experienced an asthma attack were regularly monitored in the asthma outpatient clinic. However, one patient with a first wheezing episode who responded well to treatment with bronchodilators and steroids for hMPV-LRTI monoinfection tested negative in allergy skin prick testing, and the asthma diagnosis remained unconfirmed by provocation testing.

Understanding the molecular pathways of hMPV infection is crucial for comprehending its clinical implications. In animal models of acute hMPV infection, significant airway obstruction and hyper-responsiveness persisted for at least 70 days after infection [25]. A study on BALB/c mice inoculated with hMPV type A revealed the persistence of hMPV RNA and pulmonary inflammation five months after infection. Their findings showed that hMPV-infected mice exhibited a tall and hypertrophic respiratory epithelium and a clinically significant increase in resting respiratory rate. Initially, interstitial inflammation and alveolitis were observed during the first 2–3 weeks, followed by peribronchiolar and perivascular infiltrates, which remained significant until day 154 post infection. The authors concluded that acute hMPV infection in BALB/c mice is associated with long-term lung inflammation, leading to significant obstructive airway disease. Numerous studies have elucidated the molecular pathways of hMPV infection, finding that hMPV directly infects airway epithelial cells, leading to necrosis, an inflammatory neutrophil response, and increased mucus production [26,27,28,29]. Recent investigations have also highlighted the formation of actin-based filamentous structures by hMPV, which are crucial for viral spread by facilitating cell-to-cell transmission and evading neutralizing antibodies [30]. Additionally, hMPV can impair both innate and adaptive immune responses. Its *p* protein inhibits interferon-I production, while CD4+ T lymphocyte activation is compromised during infection. Moreover, neutralizing antibody capacities are limited. This suggests a profound interaction between hMPV and the host’s immune system, potentially perpetuating inflammation due to the virus’s low replication rates in tissues [31]. Recent studies show that although hMPV peaks 48 h after infection, residual infection persists for up to 144 h, highlighting its ability to persist in the host [30]. A recent paper proposed a mechanism that elucidates how hMPV exacerbates asthma by infecting the respiratory epithelium, which triggers an antiviral response and the production of IFN-I [32]. This, in turn, activates mucosal dendritic cells, initiating a Th2 signaling cascade. In individuals with asthma, exposure to allergens exacerbates this response, leading to heightened airway inflammation and impaired viral clearance due to increased production and secretion of IL-4, IL-5, and IL-13. In our study, patients with hMPV LRTI exhibited higher median C-reactive protein levels than those with URTI. However, we observed no significant difference in the levels of inflammatory markers between patients with and without hMPV LRTI-associated hypoxemia.

Our study’s limitations include the recruitment of patients solely from university hospitals, potentially biasing the sample towards more severe hMPV cases. The retrospective design of our study constrained data collection to the most commonly used clinical variables, the majority of which were consistently recorded for all patients. A prospective design would have allowed for the assessment of additional measures of inflammation and disease severity, providing a more comprehensive understanding of the pathogenic role of hMPV. Nevertheless, it is essential to note that few clinical studies to date have delved into the connection between asthma exacerbation, a history of atopy, and hMPV infection.

## 5. Conclusions

Our study underscores the importance of recognizing wheezing in children with hMPV LRTI as an indicator of potential clinical deterioration leading to hypoxic respiratory failure, necessitating oxygen supplementation and anti-inflammatory interventions. Our findings also imply a possible heightened inflammatory response in hMPV-infected patients with a history of atopy or asthma, emphasizing the need for careful management in this vulnerable population. These insights underscore the necessity of tailored approaches to treatment and vigilant monitoring to mitigate the risk of adverse outcomes in affected children. Moreover, as suggested by long-term studies, the enduring impact of hMPV on respiratory health highlights the need for continued research to unravel the intricate mechanisms underlying hMPV pathogenesis and its implications for pediatric respiratory health.

## Figures and Tables

**Table 1:** Characteristics of children with PCR-positive hMPV infection presenting as upper or lower respiratory tract infection. Significant differences (*p* < 0.05) are highlighted in bold.

Characteristic	hMPV URTI (*n* = 28)	hMPV LRTI (*n* = 50)	Test Statistic, *p*-Value
Age (y), mean (SD)	4.2 (SD 3.7)	2.5 (SD 2.4)	t = 2.172, **0.036**
Male, no. (%)	17 (61%)	30 (60%)	χ2 = 0.004, 0.951
Chronic disease, no. (%)	8 (29%)	1 (2%)	χ2 = 12.415, **<0.001**
Viral co-infection, no. (%)	14 (50%)	19 (38%)	χ2 = 1.059, 0.303
Atopy, no. (%)	1 (4%)	4 (8%)	χ2 = 0.587, 0.444
Prematurity, no. (%)	0 (0%)	3 (6%)	χ2 = 1.747, 0.186
Disease presentation			
Fever, no. (%)	19 (68%)	41 (82%)	χ2 = 2.022, 0.155
Fever duration (days), mean (SD)	4 (SD 2)	5 (SD 3)	t = -0.924, 0.359
Rhinorrea, no. (%)	18 (64%)	43 (86%)	χ2 = 4.965, **0.026**
Cough, no. (%)	23 (82%)	49/50 (98%)	χ2 = 6.356, **0.012**
Dyspnea, no. (%)	1 (4%)	25 (50%)	χ2 = 17.411, **<0.001**
Tachypnea, no. (%)	1 (4%)	29 (58%)	χ2 = 22.465, **<0.001**
Laboratory results			
CRP (mg/L), median (IQR)	10.0 (IQR 5.0–41.0)	42.0 (IQR 10.0–89.0)	U = 857.000, **0.034**
WBC (×10^9^/L), mean (SD)	11.1 (SD 5.6)	12.9 (SD 5.3)	t = −1.457, 0.149
Bacterial pneumonia, no. (%)	3 (11%)	20 (40%)	χ2 = 7.404, **0.007**
Systemic steroids, no. (%)	0 (0%)	7 (14%)	χ2 = 4.306, **0.038**
Hospitalization, no. (%)	15 (54%)	35 (70%)	χ2 = 2.105, 0.147
Hospital stay (days), median (IQR)	2 (IQR 1–4)	4 (IQR 2–6)	U = 355.500, **0.046**
Causes of hospitalization			
Dehydration, no. (%)	3 (20%)	17 (49%)	χ2 = 3.571, 0.059
UTI, no. (%)	4 (27%)	1 (3%)	χ2 = 6.614, **0.010**
Febrile convulsions, no. (%)	2 (13%)	1 (3%)	χ2 = 2.043, 0.153
Hypoxemia, no. (%)	0 (0%)	21 (42%)	χ2 = 16.093, **<0.001**
ICU admission, no. (%)	0 (0%)	2 (4%)	χ2 = 1.149, 0.284

Abbreviations: CRP, C-reactive protein; ICU, intensive care unit; IQR, interquartile range; LRTI, lower respiratory tract infection; URTI, upper respiratory tract infection; UTI, urinary tract infection; WBC: white blood cell count. Continuous variables were compared using an independent samples T-test (test statistic *t*) or Mann–Whitney U-test (test statistic U), where appropriate, whereas categorical variables were compared by using the Pearson chi-square test (test statistic χ2).

**Table 2:** Characteristics of children with PCR-positive hMPV with and without lower respiratory tract infection-associated hypoxemia. Significant differences (*p* < 0.05) are highlighted in bold.

Characteristic	hMPV LRTI Hypoxemia (*n* = 21)	hMPV LRTI No Hypoxemia (*n* = 29)	Test Statistic, *p*-Value
Age (y), mean (SD)	2.6 (SD 2.6)	2.5 (SD 2.3)	t = −0.078, 0.938
Male, no. (%)	14 (67%)	16 (55%)	χ2 = 0.670, 0.413
Chronic condition, no. (%)	2 (10%)	1 (3%)	χ2 = 0.797, 0.372
Viral co-infection, no. (%)	7 (33%)	12 (41%)	χ2 = 0.335, 0.563
Atopy, no. (%)	4 (19%)	0 (0%)	χ2 = 6.004, **0.014**
Prematurity, no. (%)	2 (10%)	1 (3%)	χ2 = 0.797, 0.372
Fever, no. (%)	17 (81%)	24 (83%)	χ2 = 0.027, 0.870
Fever duration (days), mean (SD)	5 (SD 3)	5 (SD 3)	t = 0.033, 0.974
Dyspnea, no. (%)	18 (86%)	7 (24%)	χ2 = 18.473, **<0.001**
Tachypnea, no. (%)	19 (90%)	10 (34%)	χ2 = 15.676, **<0.001**
Asthma attack, no. (%)	5 (24%)	0 (0%)	χ2 = 7.672, **0.006**
Lung auscultation			
Wheezing, (%)	57%	31%	χ2 = 3.408, 0.065
Unilateral/bilateral crackles, (%)	5%/86%	36%/54%	χ2 = 6.797, **0.031**
CRP (mg/L), median (IQR)	29.0 (IQR 10.0–59.5)	56.0 (IQR 8.3–105.5)	U = 240.500, 0.279
WBC (×10^9^/L), mean (SD)	11.1 (SD 4.5)	14.2 (SD 5.6)	t = 2.133, **0.038**
X-ray findings			
Infiltrates, no. (%)	15/16 (94%)	15/16 (94%)	χ2 = 0.000, 1.000
Effusion, no. (%)	2/16 (13%)	2/16 (13%)	χ2 = 0.000, 1.000
Bacterial pneumonia, no. (%)	9 (43%)	11 (38%)	χ2 = 0.123, 0.726
Systemic steroids, no. (%)	6 (29%)	1 (3%)	χ2 = 6.385, **0.012**
Hospitalization, no. (%)	21 (100%)	14 (48%)	χ2 = 15.517, **<0.001**
Hospital stay (days), median (IQR)	4 (IQR 3–7)	2 (IQR 2–5)	U = 200.000, 0.077
ICU admission, no. (%)	1 (5%)	1 (3%)	χ2 = 0.055, 0.815

Abbreviations: CRP, C-reactive protein; ICU, intensive care unit; IQR, interquartile range; LRTI, lower respiratory tract infection; URTI, upper respiratory tract infection; UTI, urinary tract infection; WBC: white blood cell count. Continuous variables were compared using an independent samples T-test (test statistic *t*) or Mann–Whitney U-test (test statistic U), where appropriate, whereas categorical variables were compared by using the Pearson chi-square test (test statistic χ2).

**Table 3:** Characteristics of children with PCR-positive hMPV monoinfection with and without lower respiratory tract infection-associated hypoxemia. Significant differences (*p* < 0.05) are highlighted in bold.

Characteristic	hMPV LRTI Hypoxemia No Viral Codetection (*n* = 14)	hMPV LRTI No Hypoxemia No Viral Codetection (*n* = 17)	Test Statistic, *p*-Value
Age (y), mean (SD)	2.9 (SD 2.9)	3.1 (SD 2.6)	t = 0.189, 0.851
Male, no. (%)	8 (57%)	12 (71%)	χ2 = 0.606, 0.436
Chronic condition, no. (%)	1 (7%)	1 (6%)	χ2 = 0.020, 0.887
Atopy, no. (%)	3 (21%)	0 (0%)	χ2 = 4.033, **0.045**
Prematurity, no. (%)	1 (7%)	1 (6%)	χ2 = 0.020, 0.887
Fever, no. (%)	11 (79%)	14 (82%)	χ2 = 0.070, 0.791
Fever duration (days), mean (SD)	5 (SD 3)	4 (SD 3)	t = −0.507, 0.617
Dyspnea, no. (%)	12 (86%)	3 (18%)	χ2 = 14.243, **<0.001**
Tachypnea, no. (%)	13 (93%)	5 (29%)	χ2 = 12.692, **<0.001**
Asthma attack, no. (%)	3 (21%)	0 (0%)	χ2 = 4.033, **0.045**
Lung auscultation			
Wheezing, (%)	64%	12%	χ2 = 9.251, **0.002**
Unilateral/bilateral crackles, (%)	7%/86%	56%/38%	χ2 = 8.304, **0.016**
CRP (mg/L), median (IQR)	34.5 (IQR 5.8–82.3)	33.0 (IQR 9.0–112.0)	U = 100.000, 0.468
WBC (×10^9^/L), mean (SD)	10.6 (SD 4.4)	14.7 (SD 6.5)	t = 2.023, 0.052
Bacterial pneumonia, no. (%)	6 (43%)	9 (53%)	χ2 = 0.313, 0.576
Systemic steroids, no. (%)	4 (29%)	0 (0%)	χ2 = 5.577, **0.018**
Hospitalization, no. (%)	14 (100%)	11 (65%)	χ2 = 6.127, **0.013**
Hospital stay (days), median (IQR)	5 (IQR 4–8)	2 (IQR 2–5)	U = 109.500, 0.075
ICU admission, no. (%)	1 (7%)	1 (6%)	χ2 = 0.020, 0.887

Abbreviations: CRP, C-reactive protein; ICU, intensive care unit; IQR, interquartile range; LRTI, lower respiratory tract infection; URTI, upper respiratory tract infection; UTI, urinary tract infection; WBC: white blood cell count. Continuous variables were compared using an independent samples T-test (test statistic *t*) or Mann–Whitney U-test (Test statistic U), where appropriate, whereas categorical variables were compared by using the Pearson chi-square test (test statistic χ2).

**Table 4:** Multiple logistic regression analysis for hypoxemic hMPV lower respiratory tract infection monoinfection in children. Significant differences (*p* < 0.05) are highlighted in bold.

	*p*-Value	Odds Ratio (OR)	95% CI for OR
Age	0.329	1.31	0.77–2.22
Gender	0.347	2.80	0.33–23.89
Wheezing	**0.026**	10.32	1.33–80.40
Bilateral crackles	0.111	7.11	0.64–79.29

Abbreviations: CI, confidence interval.

## Data Availability

Data are contained within the article.

## References

[1] Panda S., Mohakud N.K., Pena L., Kumar S. (2014). Human metapneumovirus: Review of an important respiratory pathogen. Int. J. Infect. Dis..

[2] Van den Hoogen B.G., de Jong J.C., Groen J., Kuiken T., de Groot R., Fouchier R.A., Osterhaus A.D. (2001). A newly discovered human pneumovirus isolated from young children with respiratory tract disease. Nat. Med..

[3] Cattoir L., Vankeerberghen A., Boel A., Van Vaerenbergh K., De Beenhouwer H. (2019). Epidemiology of RSV and hMPV in Belgium: A 10-year follow-up. Acta Clin. Belg..

[4] Van den Hoogen B.G., Van Doornum G.J.J., Fockens J.C., Cornelissen J.J., Beyer W.E.P., De Groot R., Osterhaus A.D., Fouchier R.A. (2003). Prevalence and Clinical Symptoms of Human Metapneumovirus Infection in Hospitalized Patients. J. Infect. Dis..

[5] Yi L., Zou L., Peng J., Yu J., Song Y., Liang L., Guo Q., Kang M., Ke C., Song T. (2019). Epidemiology, evolution and transmission of human metapneumovirus in Guangzhou China, 2013–2017. Sci. Rep..

[6] Schildgen V., van den Hoogen B., Fouchier R., Tripp R.A., Alvarez R., Manoha C., Williams J., Schildgen O. (2011). Human metapneumovirus: Lessons learned over the first decade. Clin. Microbiol. Rev..

[7] Edwards K.M., Zhu Y., Griffin M.R., Weinberg G.A., Hall C.B., Szilagyi P.G., Staat M.A., Iwane M., Prill M.M., Williams J.V. (2013). Burden of Human Metapneumovirus Infection in Young Children. N. Engl. J. Med..

[8] Esposito S., Mastrolia M.V. (2016). Metapneumovirus Infections and Respiratory Complications. Semin. Respir. Crit. Care Med..

[9] García-García M.L., Calvo C., Casas I., Bracamonte T., Rellán A., Gozalo F., Tenorio T., Pérez-Breña P. (2007). Human metapneumovirus bronchiolitis in infancy is an important risk factor for asthma at age 5. Pediatr. Pulmonol..

[10] Vicente D., Montes M., Cilla G., Pérez-Trallero E. (2004). Human metapneumovirus and chronic obstructive pulmonary disease. Emerg. Infect. Dis..

[11] Perchetti G.A., Wilcox N., Chu H.Y., Katz J., Khatry S.K., LeClerq S.C., Tielsch J.M., Jerome K.R., Englund J.A., Kuypers J. (2021). Human Metapneumovirus Infection and Genotyping of Infants in Rural Nepal. J. Pediatr. Infect. Dis. Soc..

[12] Gaillard E.A., Kuehni C.E., Turner S., Goutaki M., Holden K.A., de Jong C.C.M., Lex C., Lo D.K.H., Lucas J.S., Midulla F. (2021). European Respiratory Society clinical practice guidelines for the diagnosis of asthma in children aged 5-16 years. Eur. Respir. J..

[13] Jevšnik Virant M., Uršič T., Kogoj R., Korva M., Petrovec M., Avšič-Županc T. (2022). Evaluation of Two Broadly Used Commercial Methods for Detection of Respiratory Viruses with a Recently Added New Target for Detection of SARS-CoV-2. Viruses.

[14] Boivin G., De Serres G., Côté S., Gilca R., Abed Y., Rochette L., Bergeron M.G., Déry P. (2003). Human Metapneumovirus Infections in Hospitalized Children. Emerg. Infect. Dis..

[15] Matsuda S., Nakamura M., Hirano E., Kiyota N., Omura T., Suzuki Y., Noda M., Kimura H. (2013). Characteristics of Human Metapneumovirus Infection Prevailing in Hospital Wards Housing Patients with Severe Disabilities. Jpn. J. Infect. Dis..

[16] Williams J.V., Harris P.A., Tollefson S.J., Halburnt-Rush L.L., Pingsterhaus J.M., Edwards K.M., Wright P.F., Crowe J.E. (2004). Human Metapneumovirus and Lower Respiratory Tract Disease in Otherwise Healthy Infants and Children. N. Engl. J. Med..

[17] Sobkowiak P., Mikoś M., Bręborowicz A., Szczepankiewicz A. (2020). Human bocavirus and metapneumovirus in acute wheezing in children—Is there a link with atopy?. Clin. Respir. J..

[18] Jackson D.J., Gangnon R.E., Evans M.D., Roberg K.A., Anderson E.L., Pappas T.E., Printz M.C., Lee W.M., Shult P.A., Reisdorf E. (2008). Wheezing rhinovirus illnesses in early life predict asthma development in high-risk children. Am. J. Respir. Crit..

[19] Bedolla-Barajas M., Montero H., Morales-Romero J., Landa-Cardeña A., Díaz J., Delgado-Figueroa N., Orozco-Alatorre L.G. (2017). Prevalence of respiratory viruses in wheezing children not older than 24 months of age. Gac. Med. Mex..

[20] Kusel M.M.H., de Klerk N.H., Kebadze T., Vohma V., Holt P.G., Johnston S.L., Sly P.D. (2007). Early-life respiratory viral infections, atopic sensitization, and risk of subsequent development of persistent asthma. J. Allergy Clin. Immunol..

[21] Williams J.V., Crowe J.E., Enriquez R., Minton P., Peebles R.S., Hamilton R.G., Higgins S., Griffin M., Hartert T.V. (2005). Human metapneumovirus infection plays an etiologic role in acute asthma exacerbations requiring hospitalization in adults. J. Infect. Dis..

[22] Williams J.V., Tollefson S.J., Heymann P.W., Carper H.T., Patrie J., Crowe J.E. (2005). Human metapneumovirus infection in children hospitalized for wheezing. J. Allergy Clin. Immunol..

[23] Furuta T., Hasegawa S., Mizutani M., Iwai T., Ohbuchi N., Kawano S., Tashiro N., Uchida M., Hasegawa M., Motoyama M. (2018). Burden of Human Metapneumovirus and Respiratory Syncytial Virus Infections in Asthmatic Children. Pediatr. Infect. Dis. J..

[24] Taniguchi A., Kawada J.I., Go K., Fujishiro N., Hosokawa Y., Maki Y., Sugiyama Y., Suzuki M., Tsuji T., Hoshino S. (2019). Comparison of Clinical Characteristics of Human Metapneumovirus and Respiratory Syncytial Virus Infections in Hospitalized Young Children. Jpn. J. Infect. Dis..

[25] Hamelin M.E.V., Prince G.A., Gomez A.M., Kinkead R., Boivin G. (2006). Human Metapneumovirus Infection Induces Long-Term Pulmonary Inflammation Associated with Airway Obstruction and Hyperresponsiveness in Mice. J. Infect. Dis..

[26] Kuiken T., van Den Hoogen B.G., Van Riel D.A.J., Laman J.D., Van Amerongen G., Sprong L., Fouchier R.A., Osterhaus A.D. (2004). Experimental Human Metapneumovirus Infection of Cynomolgus Macaques (*Macaca fascicularis*) Results in Virus Replication in Ciliated Epithelial Cells and Pneumocytes with Associated Lesions throughout the Respiratory Tract. Am. J. Pathol..

[27] Williams J.V., Tollefson S.J., Johnson J.E., Crowe J.E. (2005). The Cotton Rat (*Sigmodon hispidus*) Is a Permissive Small Animal Model of Human Metapneumovirus Infection, Pathogenesis, and Protective Immunity. J. Virol..

[28] Wyde P.R., Chetty S.N., Jewell A.M., Schoonover S.L., Piedra P.A. (2005). Development of a cotton rat-human metapneumovirus (hMPV) model for identifying and evaluating potential hMPV antivirals and vaccines. Antivir. Res..

[29] Vargas S.O., Kozakewich H.P.W., Perez-Atayde A.R., McAdam A.J. (2004). Pathology of human metapneumovirus infection: Insights into the pathogenesis of a newly identified respiratory virus. Pediatr. Dev. Pathol..

[30] Kinder J.T., Moncman C.L., Barrett C., Jin H., Kallewaard N., Dutch R.E. (2020). Respiratory Syncytial Virus and Human Metapneumovirus Infections in Three-Dimensional Human Airway Tissues Expose an Interesting Dichotomy in Viral Replication, Spread, and Inhibition by Neutralizing Antibodies. J. Virol..

[31] Gálvez N.M.S., Andrade C.A., Pacheco G.A., Soto J.A., Stranger V., Rivera T., Vásquez A.E., Kalergis A.M. (2021). Host components that modulate the disease caused by hMPV. Viruses.

[32] Rudd P.A., Thomas B.J., Zaid A., MacDonald M., Kan-O K., Rolph M.S., Soorneedi A.R., Bardin P.G., Mahalingam S. (2017). Role of human metapneumovirus and respiratory syncytial virus in asthma exacerbations: Where are we now?. Clin. Sci..

